# A systematic review and narrative synthesis of fathers’ (including migrant fathers’) experiences of pregnancy and childbirth

**DOI:** 10.1186/s12884-023-05568-8

**Published:** 2023-04-11

**Authors:** Andy Mprah, Melanie Haith-Cooper, Eva Duda-Mikulin, Fiona Meddings

**Affiliations:** 1 Faculty of Health Studies, University of Bradford, Richmond Road, Bradford, BD7 1DP UK; 2 Centre for Interdisciplinary Research into Health and Illness, University of Wroclaw, Wroclaw, Poland; 3 Faculty of Health Sciences, University of York, Seebohm Rowntree Building, Heslington, York, YO10 5DD UK

**Keywords:** Narrative synthesis, Childbirth, Pregnancy, Experience, Fatherhood, Father, Migrant father

## Abstract

**Objective:**

The purpose of this review was to consider factors that influence the experiences of pregnancy and childbirth by fathers including migrant fathers.

**Method:**

A systematic review and narrative synthesis were conducted as per the PRISMA guidelines. The spider tool was used to build a search strategy which was used to conduct literature search in eight identified electronic databases: ASSIA, CINAHL, EMBASE, MEDLINE, PsycINFO, PUBMED, Sage and Scopus. Grey literature was searched through the King’s Fund Library database, Ethos, The North Grey Literature Collection, Social Care Online and other charity websites such as the Refugee Council and Joseph Rowntree Foundation. The search was conducted across all the databases in the week commencing January 7, 2019, and restricted to studies published in the English language.

**Results:**

The search across all the eight electronic databases identified 2564 records, 13 records through grey literature databases/websites and an additional 23 records identified through hand-searching/forward citation. The number of records after duplicates were removed was 2229. Record screening based on titles and abstracts identified 69 records for full text screening. Dual screening of these full text records identified 12 full records from 12 separate studies, eight of which were qualitative studies, three of which were quantitative studies and one mixed method study.

**Findings:**

This review has revealed three main themes: influence of society and health professionals; adjustment to a new life of fatherhood; and involvement in maternity care. However, the literature has focused on non-migrant father’s experiences of pregnancy and childbirth, with little attention paid to fathers who may be migrants.

**Key conclusion and implications for practice:**

This review has exposed a dearth of research on migrant fathers’ experiences of pregnancy and childbirth in an era of increasing globalisation and migration between countries. Midwives and other health professionals should be alert to the needs of any father when providing maternity care. More research is needed which considers experiences of migrants and how choosing to move to a new country or being forced to move could influence migrant father’s experiences and therefore their needs.

**Supplementary Information:**

The online version contains supplementary material available at 10.1186/s12884-023-05568-8.

## Introduction

It is important to understand men’s experiences of pregnancy and childbirth, as co-users of maternity services. Ensuring a positive experience for men, as supporters of their pregnant partners will influence women’s experiences [1]. It is even more important to understand the pregnancy and childbirth experience of migrant men who have moved to a new country with possibly a very different healthcare system. Most research in the area of father’s engagement with pregnancy and childbirth has focused on white middle-class men [2]. Migrant fathers’ experiences need exploring as they may have a challenging experience when interacting with maternity care, particularly because of potential language barriers, social isolation and discrimination, lack of knowledge of different midwifery practices and cultural differences [3–7]. Given that in many countries, there is a rapid growth in the population of migrant families with children [8], it is important to consider the needs of migrant fathers in the context of maternity care.

It is estimated that one-seventh of the world’s population now live in a different country to where they were born [8]. It is also estimated worldwide that large numbers of migrant women are of childbearing age and therefore maternity service users [9]. Most high-income countries like the UK have experienced significant levels of both planned and forced migration in the last few decades, resulting in an increased ethno-cultural diversity [10] which has impacted on health care delivery. In the UK, lower maternal mortality rates (MMR) were recorded among indigenous whites (9 per 100,000 maternities) compared to Indian migrants (20.5), Pakistani migrants (13.9), Bangladeshi migrants (11.1), Caribbean (18.5) and 26.9 among African migrants [11]. There were similar trends in the USA [12, 13], the Netherlands [14] and Australia [15, 16]. The expectations of the migrant may vary between different geographical and cultural locations [17]. Poor maternal outcomes may influence the migrant mother, the baby [18, 19] but also, the migrant father who may choose to support his partner and is a co-user of maternity services.

There are different types of migrants. These includes asylum seekers, economic migrants and transients [20] who are temporary migrants with limited visas [21]. Migrants have also been classified as undocumented, forced, free, controlled and ‘illegal’ [8]. Several factors such as economic improvement, family reunion and seeking refugee status have served as motivation for migration. For this paper, migrants will be classified as either economic migrants or forced migrants. Of all migrants, forced migrants (refugees and asylum seekers) have received the most attention in both academic and policy terms [22]. This is because they may have particularly challenging migration trajectories. They may have suffered abuse and trauma during migration compared to other migrants, and they may be exposed to unfavourable conditions in their receiving countries including marginalisation in maternity services. Being a forced migrant increases the risk of marginalisation in maternity services [6, 23, 24]. Hence, the need for greater understanding of the experiences of refugee, asylum-seeking and undocumented migrant families to inform health, social services, practices and policies [25].

There have been reported inequalities in access to maternity care between migrant women and non-migrant women [26]. International research has demonstrated that many migrant women struggle accessing maternity care in the receiving country and experience poorer maternal health outcomes than the non-migrant women [5, 27–29]. In addition, migrant fathers may experience stress being fathers in a country different to their home country. The way fatherhood is socially constructed may be different in the father’s home country when compared to the host country. Therefore, the man may have different expectations in relation to his role during pregnancy [30, 31].

Ethnic discrimination has been cited as one important source of stress [32, 33] which may be experienced by migrant mothers but also fathers. Stress exposure in varying forms has been cited as key in health inequalities [34, 35]. The ultimate outcome of this is a pathological effect on the migrant mother and the baby [18, 19] and it could also affect the migrant father. Stress is linked with adverse birth outcomes including very low birth weight babies [36–39], preterm birth, developmental delays and behavioural abnormalities in children [40–42]. It is not clear how stress influences the migrant father and research is needed to understand migrant fathers’ experiences of maternity care to ensure their needs are met. In addition, there is the need to bring migrant fathers’ experiences of pregnancy and childbirth more into focus [43] due to the potential mismatch between the expectations of the father during pregnancy and childbirth in home country and in the host country.

Although there is a wealth of literature related to migrant maternal experiences [3, 44], an initial scoping exercise found no studies could be found that focus directly on migrant fathers’ experiences of pregnancy and childbirth. Most of the studies refer to fathers more generally [45–51]. As such, it is important to consider migrant fathers within this larger population through a systematic review of current studies.

This paper, therefore, reports on a systematic review undertaken to explore fathers’ experiences of pregnancy and childbirth generally, so that data related to migrant fathers can be extracted and used to inform maternity care.

### Review question

What are fathers’ experiences of pregnancy and childbirth?

### Sub-question

What are migrant fathers’ experiences of pregnancy and childbirth?

## Methods

This review was conducted in accordance with recommendations outlined in the PRISMA (Preferred Reporting Items for Systematic Reviews and Meta-analyses) statement [52]. It was undertaken adopting a narrative synthesis framework [53]. The review protocol was registered with the International prospective register of systematic reviews PROSPERO with reference CRD42019127792.

### Search strategy

This was designed in conjunction with an experienced health studies librarian. In January 2019, eight identified electronic databases: ASSIA, CINAHL, EMBASE, MEDLINE, PsycINFO, PUBMED, Sage and Scopus were searched to identify eligible studies based on our search criteria. Grey literature was searched through the King’s Fund Library database, Ethos, The North Grey Literature Collection, Social Care Online and other charity websites such as the Refugee Council and Joseph Rowntree Foundation. The SPIDER tool [54] was used to identify search terms (see Table 1). These respective Medical Subject Headings (MeSH) terms were used including the Boolean terms “OR”/ “AND,” and truncation.

**Table 1:** Search terms using the SPIDER Tool

SPIDER	SPIDER Tool	Search Terms
Sample	S	“fathers” OR “men” OR “paternal” or “migrant fathers” OR “migrant men” OR “economic migrant fathers” OR “forced migrant fathers” OR “international migrant fathers” OR " undocumented migrant fathers” OR “documented migrant fathers” OR “educational migrant fathers” OR “transients” OR “refugee fathers” OR “asylum seeker fathers” OR “first generation migrants” OR “second generation migrants” OR “regular migrant fathers " OR (im) migrants OR “minority”
Phenomenon of Interest	P of I	“pregnancy experience” OR “pregnancy outcomes” OR “maternal experiences” “paternal experiences” OR “maternal health outcomes” OR “perinatal period” OR “antenatal period” OR “postnatal period” OR “postpartum period”
Design	D	“questionnaire*” OR “survey*” OR “interview*” OR “focus group*” OR “case study*” OR “observ*”
Evaluation	E	“view*” OR “experienc*” OR “opinion*” OR “attitude*” OR “perce*” OR “belie*” OR “feel*” OR “know*” OR “understand*”
Research Type	R	“qualitative” OR “quantitative” OR “mixed methods”

Included studies were all research designs, published from 2009 to 2021, in the English language. Studies were based on primary research focusing on fathers, exploring their experiences in pregnancy and up to six weeks following birth in high income countries, as defined by the World Bank [55]. All participants included in this review were males aged between 18 and 44 years.

### Study selection

**Fig. 1:**
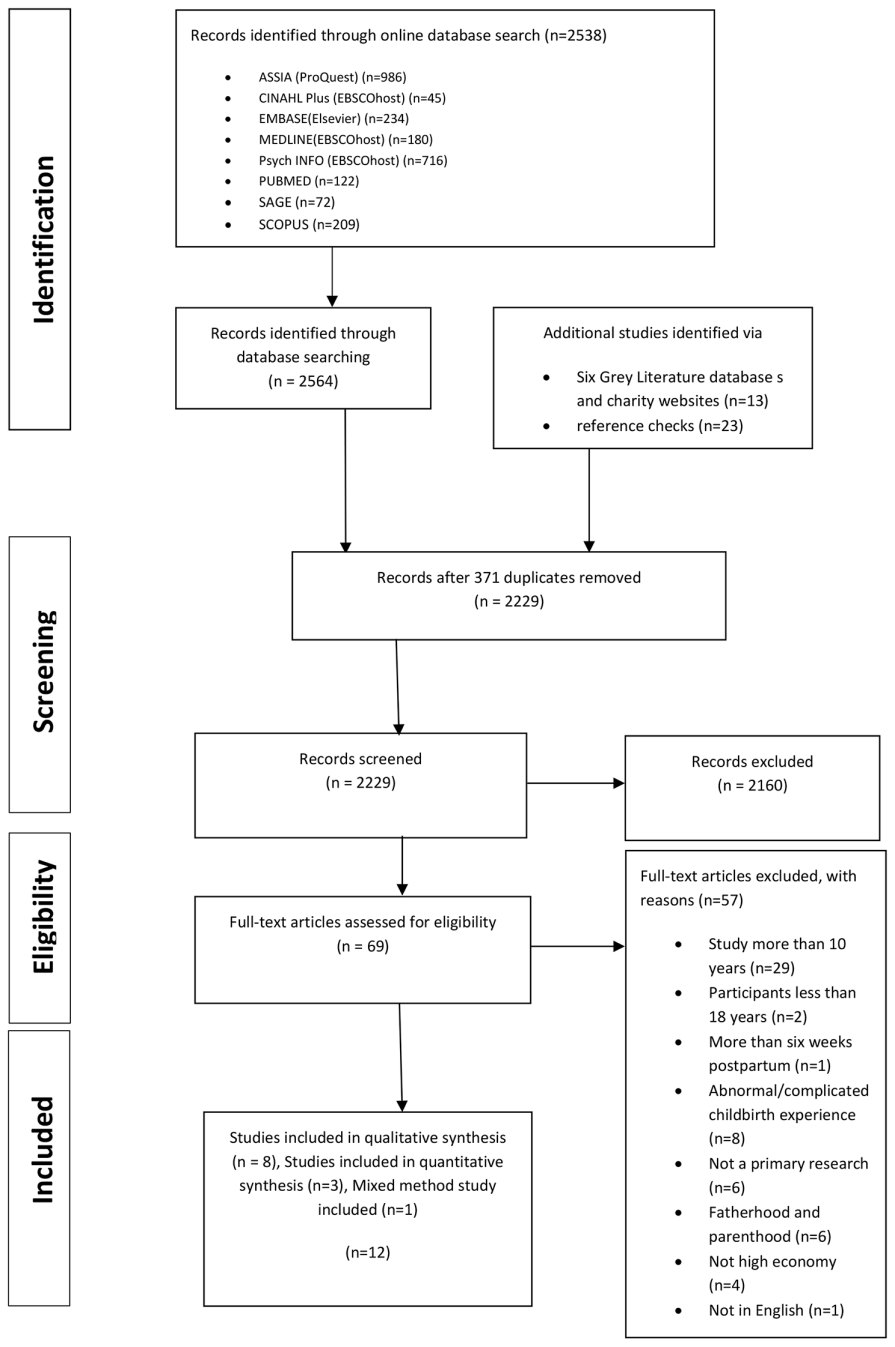
PRISMA flow diagram for the systematic literature search

The electronic search identified 2564 records, 13 records through grey literature databases/websites and an additional 23 records identified through hand-searching. After duplicates were removed, 2229 remained. Screening based on titles and abstracts identified 69 records. Screening of these full text records identified 12 papers from 12 separate studies, eight of which were qualitative studies, three quantitative and one mixed method studies (see Fig. 1).

### Quality appraisal and data extraction

All study details including study aims, participant details, study settings, methods of data collection, findings, results and analyses were initially extracted using standardised data extraction forms for qualitative [56], quantitative [57] and a self-devised mixed-method data extraction form.

The relevant data from each study was extracted including study aims, participant details, study settings, methods of data collection, findings, results, and analyses. A self-devised mixed-method data extraction form was used for the mixed methods study, created by merging the qualitative [56] and quantitative [57] data extraction forms. Data extraction was checked individually by the co-authors and discrepancies resolved as a group. We adopted the QATSDD tool, a 16-item quality assessment tool [58] to appraise the quality all study designs. Criterion for each respective study was scored between 0 and 3.

The percentage of maximum quality score for each study was calculated by dividing the respective total quality score by the maximum score each study could have scored and multiplied by 100 (See Table 2). The methodological quality of the studies included in this review mostly ranged from high to medium, except for one study that scored low [59]. No study was excluded based on quality.

**Table 2:** Quality score and percentage of maximum quality score of each included study

	INCLUDED EMPIRICAL STUDIES (SCORE- 1–3)																							
QUALITY CRITERIA	[60]		[61]	[59]		[62]		[63]			[64]		[65]		[66]	[67]			[68]		[69]		[70]	
Explicit theoretical framework	3		2	1		3		1			1		1		2	2			2		3		3	
Aims and objectives stated in body	3		3	3		3		3			3		3		3	3			3		3		3	
Clear setting description	3		3	3		3		3			3		3		3	3			3		3		3	
Sample size considered in terms of analysis	3		3	2		1		3			3		3		3	2			3		3		2	
Representative sample of target group of a reasonable size	1		2	2		2		3			3		3		3	2			3		2		2	
Data collection procedure description	3		3	2		3		3			3		3		3	3			3		3		3	
Data collection tool rationale	3		3	2		3		3			3		3		3	3			3		3		3	
Detailed recruitment data	3		3	2		3		3			3		3		3	3			3		3		3	
Statistical assessment of reliability and validity of measurement tool(s)	-		-	-		-		3			-		3		3	-			3		-		-	
Fit between stated research question and data collection method (Quantitative)	-		-	-		-		3			-		3		3	-			3		-		-	
Fit between stated research question and format/content of data collection tool (Qualitative)	3		3	3		3		-			3		3		-	3			-		3		3	
Fit between research question and method of analysis	3	3			3		3		3	3		3		3			3			3		3		3
Good justification for analytical method	3	3			2		3		3	3		3		2			3			3		3		3
Assessment of reliability of analytical process (Qualitative only)	3	2			3		3		-	3		1		-			3			-		3		2
Evidence of user involvement in design	3	3			2		3		3	1		2		2			3			2		2		2
Strengths and limitations discussed critically	3	1			0		1		3	3		3		1			3			3		2		3
**Total quality score for each study**	40	37			30		37		40	38		43		37			36			40		39		38
**Percentage of maximum quality score (%)**	**95.2**	**88.1**			**71.4**		**88.1**		**95.2**	**90.5**		**89.6**		**88.1**			**85.7**			**95.2**		**92.9**		**90.5**

Adapted from Sirriyeh, Lawton [58].

**KEY TO SCORES**:

**0 = Not at all**

**1 = Very slightly**

**2 = Moderately**

**3 = Completely**

The stages of this synthesis were conducted iteratively using the appropriate elements of Popay, Roberts [53] toolbox. These are to develop a theory, to develop a preliminary synthesis, explore relationships between/within studies and assess the robustness of the synthesis process.

The characteristics of the included studies are as shown in Table 3.

**Table 3:** Characteristics of included studies

STUDY, COUNTRY	STUDY TITLE	STUDY AIM	NO. OF FATHERS (NO. OF MIGRANT FATHERS)	STUDY DESIGN	RESULTS
[60], **USA**	A community perspective on the role of fathers during pregnancy: A qualitative study	The primary objective of the focus groups was to obtain mothers’ and fathers’ thoughts on the role of the father during their partner’s pregnancy to inform next steps of the National Healthy Start Association’s fatherhood initiative.	13(0)	Community-based participatory approach, focus groups, content and thematic analysis	In this study, an involved father during pregnancy is defined by participants as being accessible (present or available); engaged (cares about the pregnancy and wants to learn more about the process); responsible (caregiver, provider, protector); and maintaining relationship with the woman carrying the child, regardless of their own partnership status.
[61], **SWEDEN**	Support during labour: first-time fathers’ descriptions of requested and received support during the birth of their child	The aim of this study was to explore how first-time fathers describe requested and received support during a normal birth, it was decided that individual interviews was the most suitable method to understand their descriptions	10(0)	Open-ended interviews, latent content analysis	The support described is presented as one main theme, ‘being involved or being left out’, which included four underlying categories: ‘an allowing atmosphere’, ‘balancing involvement’, ‘being seen’ and ‘feeling left out’.
[59], **UK**	A qualitative exploration of first-time fathers’ experiences of becoming a father	To explore first-time fathers’ experiences of becoming a father, focusing on their expectations and experiences, and their views on how they are coping with this transition.	9(0)	Semi-structured interviews and interpretive phenomenological analysis.	One overarching superordinate theme derived from the analytic process was ‘searching for a place’, which reflected the process in which fathers searched for their role and position in relation to their partner and child
[62], **AUSTRALIA**	A qualitative investigation into the pregnancy experiences and childbirth expectations of Australian fathers-to-be	To explore and describe men’s experiences of pregnancy and childbirth expectations.	12(0)	Qualitative descriptive design and thematic analysis.	Five themes emerged: Pregnancy news heralds profound change: adjusting to pregnancy and working to see things differently; birth looming; feeling side-lined in antenatal visits; men’s childbirth expectations.
[63], **SWEDEN**	Fathers’ birth experience in relation to midwifery care	The aim was to identify the proportion of fathers having a positive experience of a normal birth and to explore factors related to midwifery care that were associated with a positive experience.	595(0)	Longitudinal survey, questionnaires, descriptive statistics and odd ratios.	The majority of fathers (82%) reported a positive birth experience. The strongest factors associated with a positive birth experience were midwife support (OR 4.0; 95 CI 2.0—8.1), the midwife’s ongoing presence in the delivery room (OR 2.0; 1.1—3.9), and information about the progress of labour (OR 3.1; 1.6—5.8).
[64], **NEW ZEALAND**	First-time fathers’ perception of their childbirth experiences	This research seeks to provide more evidence about the importance of the role of first-time fathers and provide some reflection on their experiences.	155(0)	Survey questionnaire, phenomenological thematic analysis.	Core themes included safety of mother and baby, understanding support role, mother in control, managing pain/care and communication after birth. Fathers commented on what impacted on their childbirth experiences and outlined their needs for a positive experience.
[65], **SWEDEN**	Childbirth–an emotionally demanding experience for fathers	The objective was to explore Swedish fathers’ birth experiences, and factors associated with a less-positive birth experience.	827(0)	Prospective longitudinal cohort survey, descriptive statistical analysis, content analysis.	In total, 604 (74%) of the fathers had a positive or very positive birth experience. Identified a less-positive birth experience associated with emergency caesarean section (RR 7.5; 4.1–13.6). Instrumental vaginal birth (RR 4.2; 2.3 8.0), and dissatisfaction with the partner’s medical care (RR 4.6; 2.7–7.8) were recorded.
[66], **CROATIA**	Prospective Fathers: Psychosocial Adaptation and Involvement in the Last Trimester of Pregnancy	The current study focused on expectant fathers’ perception of involvement in pregnancy during the last trimester of pregnancy that was often reported to be the most stressful time for men in their transition to parenthood.	143(0)	Questionnaire, one-way ANOVA, Correlational analysis, and hierarchical regression analysis.	The prospective fathers showed high involvement in their partner’s pregnancies, elevated levels of perceived stress and high relationship quality. There were no differences in these variables regarding complications in pregnancy and pregnancy duration.
[67], **SINGAPORE**	First-time fathers’ experiences and needs during pregnancy and childbirth: A descriptive qualitative study	To explore first-time fathers’ experiences and needs during their wives’ pregnancy and childbirth in Singapore.	16(0)	Descriptive qualitative design, semi-structured interviews and thematic analysis.	First-time fathers experienced a range of emotions from being happy and excited to feeling shocked and worried and to feeling calm. Adaptive and supportive behaviours were adopted to deal with the pregnancy changes and better support their wives.
[68], **AUSTRALIA**	An Exploration of the Perceptions of Male Partners Involved in the Birthing Experience at a Regional Australian Hospital	The aims of the current study were to document men’s self-reported perceptions of their partners’ antenatal, labor, and birthing experiences and to explore the relationships between these perceptions and men’s feelings of beneficial presence to the birthing mother	163(0)	Voluntary anonymous questionnaire and regression analysis.	There was a significant relationship between perceived benefit of the partners’ presence and positive perception of both antenatal experience and birth involvement. There also was a positive relationship between realized birth expectations and both antenatal experience and birth involvement.
[69], **SWEDEN**	First-time fathers’ experiences of childbirth—A phenomenological study	To describe fathers’ experiences during childbirth	10(2)	Phenomenological lifeworld approach, interviews guided by re-enactment method.	The four themes constituting the essence were: ‘a process into the unknown’, ‘a mutually shared experience’, ‘to guard and support the woman’ and ‘in an exposed position with hidden strong emotions.
[70], **AUSTRALIA**	Fatherhood in a New Country: A Qualitative Study Exploring the Experiences of Afghan Men and Implications for Health Services	Aimed to explore the experiences of Afghan women and men of refugee background having a baby in Melbourne, Australia.	14(14)	Community-based participatory approach, interviews/focus groups and thematic analysis.	Afghan men reported playing a major role in supporting their wives during pregnancy and postnatal care, accompanying their wives to appointments, and providing language and transport support.

The data from the included studies were organised into smaller groups to manage the synthesis process by looking for patterns within and across these groups. The themes were then grouped as shown in Table 4.

**Table 4:** Groupings and clusters of studies with respective themes

THEME AND SUB-THEMES	GROUPINGS AND CLUSTERS WITH REFERENCES FOR EACH PAPER INCLUDED
**Influence of society and health professionals**	
Outside support	Social support received [67], how helpful educators were in antenatal classes [68], competence of healthcare professionals [65], midwifery support and positive birth experience [63], An allowing atmosphere [61]
Need for information	To have the right to ask [61], current maternity care improvement [67], addressing father’s needs and concerns (14 migrant fathers) [70], how well informed male partners felt about pregnancy [68]
**Adjusting to a new life of fatherhood**	
Change in fathers’ roles	Accessibility, responsibility [60], pregnancy heralds profound change, birth looming but, adjustment to pregnancy [62], adaptive and supportive behaviour [67], emotional changes experienced [67], to guard and support the woman (2 migrant fathers) [69], supporting women during pregnancy, labour and birth (14 migrant fathers) [70], (safety of mother and baby (a. Safety as a priority, b. Healthy mother and baby) [64]
An uncharted territory	Process into the unknown (2 migrant fathers) [69], searching for a place [59], not having a clue [62], fatherhood in a new country (14 migrant fathers) [70], in an exposed position with hidden strong emotions (2 migrant fathers) [69]
**Involvement in the maternity**	
Active/passive involvement in maternity	Engagement [60], being seen [61], understanding support role [64], clearly actively engaged in pregnancy [66], mutually shared experience (2 migrant fathers) [69], care and communication after birth [64], couple relationship maintained regardless [60], support to pregnant wives/partners (14 migrant fathers) [70]
Feeling side-lined and marginalised	Feeling left-out [61], feeling side-lined [59, 62]

## Findings

This review incorporated data from 827 fathers from the mixed method study, 901 fathers across the quantitative studies and 249 fathers across the qualitative studies. Of these, 606 were first time fathers but only 16 were migrant fathers [60, 61]. Ages ranged from 18 to 52 years for all fathers and 25–43 for migrant fathers. The majority of these studies were conducted in Sweden (n = 4) [60, 62–64], followed by Australia (n = 3) [61, 65, 66] and one each in the United States of America [67], United Kingdom [59], New Zealand [68], Croatia [69] and Singapore [70]. Of all these countries, migrant fathers were only studied in Sweden (n = 2) [60] and Australia (n = 14) [61].

The narrative synthesis identified three themes; Influence of society and health professionals, adjusting to a new life of fatherhood and Involvement in the maternity care which are described below:

### Influence of society and health professionals

Six studies found that elements of society and health professionals influenced expectant fathers [60, 62, 63, 65, 66, 70]. This included a total of 1611 non-migrant fathers with 268 being first-time fathers and 14 migrant fathers.

Poh, Koh [70] reported a narrative from a non-migrant and first-time father which discussed the positive influence of the wider family on the father’s maternity experience:

Generally, it’s very useful and supportive if your parents or parents-in-law uuhh… are able to contribute as in, provide advice, share their previous experience and help you to prepare along the way. It’s a big encouragement and emotional support ah, from the family

Quantitative data highlighted that health care professionals’ competence and supportive behaviours towards the expectant non-migrant father led to a positive birth experience [63]. 82% of fathers (488 fathers) in [62], reported a positive birth experience for fathers with the strongest associated factor being midwife support (OR 4.0; 95 CI 2.0—8.1), the midwife’s ongoing presence in the delivery room (OR 2.0; 1.1—3.9), and information about the progress of labour (OR 3.1; 1.6—5.8). None of the quantitative studies included migrant fathers.

Two studies discussed how the quality of the information received about childbirth, positively influenced non-migrant fathers’ experiences. In one of these studies, the midwife was the source of such information:

*“I felt good when the midwife showed me, because then I was able to be involved’* [60].

In the other study, being involved in antenatal education was considered beneficial for the non-migrant fathers’ experiences:

*“Practice makes perfect. Hands-on la, better. Cos if the class itself is… we looked through the slides most of the time, I would say 99%. Then the rest is the baby doll, the dummy… (laughs)”* [67].

However, neither of these two studies reported the involvement of migrant fathers.

A quantitative data supported this with antenatal class attendance by most of the expectant non-migrant fathers (103, 86.6%), making them well informed about the pregnancy, childbirth process and possible complications [65].

Information from healthcare professionals was highlighted as essential for expectant fathers. The study involving only migrant fathers [66] highlighted how the health professionals supporting the migrant father also had an impact on the mother:

*“The nurses were very kind and nice and they worked very hard to serve us, I think if there was anything in the world that could be done, they would do it for us. . their support gave me good feeling and I could provide moral support to my wife”* [66].

### Experience of adjusting to a new life of fatherhood

Seven studies [59, 61, 64, 66–68, 70] discussed how fathers’ experiences of pregnancy and childbirth were influenced by their ability to adjust to fatherhood. This included their knowledge about the maternity process and also changes to social groups. A total of 213 non-migrant fathers, 195 first-time fathers and 16 migrant fathers narrated to reflect this theme.

Some non-migrant fathers discussed how they experienced an uncharted territory when faced with pregnancy and childbirth issues. This included not having a clue on what to expect from pregnancy and childbirth [61].

*“I’ve probably just blundered along following her (his partner’s) lead”* [61].

This had a negative impact on one non-migrant father’s experience:

*“You are in unknown territory ... When you’re there you know you really don’t know anything about this. I don’t know what’s going to happen … I was worried there would be no room for us; you know the worst-case scenario”* [69].

Another study [59] revealed that part of the adjustment to fatherhood included experiencing new links and memberships of different social groups One non-migrant father stated that:

*“People who are already fathers come over and have a chat with you and it is sort of a bit of a club you suddenly find yourself in”* [59].

For migrant fathers, the concept of an unknown territory was exacerbated as it involved experiencing becoming a father in a new country. Some migrant men experienced a cultural clash between the expectations of the father in their home country and the role they had to adopt in their new country. One migrant father had previous experience of childbirth in Afghanistan:

*“Actually, here all the time I was with my wife but in Afghanistan, my family, my father, mother and other relatives would take care of my wife and child, but here I play a hundred roles during pregnancy and appointments”* [70].

Another migrant father shared his experience in Australia:

*“Here in Australia, men and women have equal rights, and men are obligated to go to the hospital for every appointment, but not in Afghanistan. This is a big difference, yes”* [70].

Pregnancy led to men experiencing a profound change in their role in the household across the studies. This included some non-migrant fathers experiencing an increased sense of responsibility looking after their partners [67]. This increased responsibility included providing practical support:

*“Making sure she eats healthy, go to her doctor’s appointments. If she has to take off because of a high-risk pregnancy, he’ll maintain the bills, or other kids, if there are other kids, just whatever is needed”* [60].

Increased responsibility also included emotional support:

“*The role of a man during pregnancy is to be present, to support, to understand, to be patient, and to have sympathy for the woman carrying his child*” [60].

And getting prepared for the birth:

*We’re a lot more organised I think this time.. . I just made sure that I knew that we’d go to that door and just refreshed it…. had the car serviced... petrol’s always full, and the bag is packed and ready to go.. .* [62].

For some fathers, this increased responsibility led to the need to adapt their current life experiences to meet the needs of the mother:

We used to meet on and off during the weekends. So, I stopped going there and then even for parties, I used to attend a lot. She can’t stay there for long time. She’ll get pain… So, even if we’re going, we just go and then say hi and spend there, 10 min and come back

One study reported a non-migrant first-time father’s experience of change to his role which involved caring for his partner in a way he hadn’t before pregnancy:

“*My plan was to ensure mum and baby were kept as safe and comfortable as possible during the process*” [68].

### Involvement in the maternity experience

A quantitative study reported that most expectant fathers were actively involved in their partner’s pregnancy [69]. Eight studies found that the level of involvement by fathers in maternity care influenced how they experienced pregnancy and childbirth [59–61, 64, 66–69]. One qualitative study reported that men’s level of involvement was influenced by how close they felt to the mothers:

*“When my wife was pregnant, I was at all the appointments… so, I was there. I think it depends ... how you feel about that girl.. . [If]…you just got some random chick pregnant you’re not going to feel like going to an appointment with her, you’re not going to feel like, encouraging her”* [60].

Interestingly, one study found that fathers who initially did not want to be involved in the mother’s maternity experience changed their mind:

*“I didn’t want to be very involved beforehand, but when it all started, it was just me in there with the midwife, so I ended up getting a lot more involved than I had wanted to, but it was great”* [64].

This involvement in the maternity experience, included during labour:

*“When she was pushing at the end, I held one hand behind her neck to give her strength … and the other round her leg, so I held her together! I really felt part of it all and she said that afterwards… in that sense it felt really good. I didn’t feel left out at all!”* [69].

Migrant fathers who perhaps may not be involved with maternity in home country were also positive about the high level of involvement they had in maternity care, including during labour:

*“The fact that I was there with my wife during labour [was good], because I had never seen someone giving birth so I could understand her pain and also my wife was happy that I was there standing by her”* [70].

Another migrant father went on to state that during labour:

“*I had a big role in this. I kept her company and was always there with her to help her and support her*” [70].

Non-migrant fathers were not always actively involved in their partners maternity experience and studies reported that some non-migrant fathers felt left out when their partner was receiving maternity care [61]. “*I wanted to help, but I felt left out, I could not do anything…. I wanted to help somehow but could not…*.” [61].

Another non-migrant father reported a sense of exclusion during the labour of his partner:

*“Well obviously you’re not the priority and that’s fair enough but sometimes you feel like you’re just sort of like barely even in the room”* [62].

Feeling left out was also an issue for some non-migrant fathers during labour:

*“I felt like an absolute spare part. In all of that that went on there was nothing I could do, nothing physically… which is really weird because you want to get involved”* [59].

Some migrant fathers faced challenges with the language [66]. They could not effectively communicate their concerns and had to rely on people to translate. A migrant father stated to suggest help with language:

*“I would call someone else to get help if I didn’t understand, and try to find some way solve my problems”* [70].

## Discussion

This review examined the evidence from a total of 1977 fathers who were studied across 12 countries in 12 separate studies. However, migrant fathers’ experiences of pregnancy and childbirth were under-represented in the literature, with only 16 migrant fathers across two studies living in Australia [66] and Sweden [64].

Three themes emerged from the data exploring factors that had either a positive or negative influence on father’s experiences: influence of society and health professionals, the adjustment to a new life involving fatherhood, and involvement in maternity care. Support from family was considered positive for non-migrant fathers reflecting previous reviews but no data on this factor were available for migrant fathers, perhaps because they did not have family in their host country. Information and support from health professionals led to a positive experience for non-migrant fathers reflecting previous research [71–75]. Positive support from health professionals also had a positive influence on the experience of some of the migrant fathers [60].

Adjusting to becoming a father was a common theme across the studies with men reporting not knowing what to expect from pregnancy and childbirth. This reflected findings from a UK based survey [45]. This theme included data from migrant men (n = 16) across two different studies, who reported that difficulties adjusting to fatherhood were exacerbated by being in a new country. Some men reported cultural clashes between the expected role of a father in home country compared to their expected role in their new country.

Being involved in maternity care was considered both positive and negative for non-migrant fathers. Interestingly, this was considered positive for migrant fathers despite the cultural differences between the role of the father in the home country, where in some areas of the world, due to cultural differences, it is common to have minimal involvement in maternity care [76]. This supports previous research which highlighted cultural clashes and change in perception of fatherhood involvement by migrant African fathers in Belgium [77] and how they adjusted their native perceptions of fatherhood and fathering roles to that of western culture. Convertly, a Finnish study found there was conflict within the family when an African man was married to a white Finnish woman due to their different perceptions about the father’s role [78].

Although the number of migrant fathers was too small to draw any conclusions, there appeared to be similarities in the experiences of migrant and non-migrant fathers within the themes. However, more research is needed to explore this in detail, to consider how being a migrant in a new country may distort these experiences due to language and cultural barriers [79].

In addition, it is important to understand the heterogeneity of migrants and how factors may influence the experiences of men who originate from different areas of the world where cultural expectations of the father’s role in pregnancy and childbirth are known to vary [80, 81]. Men who have previously experienced childbirth in their home country may have a different experience of becoming a father in the new country [82]. In addition, the reason for migration may lead to different experiences, for example economic migrants, generally move to a new country voluntarily compared to men who may have been forced to migrate (asylum seekers and refugees) [83]. Forced migrants may face employment restrictions whereas economic migrants have entered a new country to work. Without adequate funds to take care of the demands of their partners’ pregnancy, forced migrant fathers’ experience could be more stressful compared to economic migrants, which could impact on their overall experience of pregnancy and childbirth. The underrepresentation of migrant men in the literature meant that the heterogeneity of the migrant father could not be adequately explored.

The findings from this review have implications for clinical practice. Midwives and other health professionals should be alert to the needs of any father when providing maternity care. Providing information to fathers and including them in episodes of care could have positive impacts on them and their partners or negative consequence when not done carefully and respectfully. Midwives and other health professionals need to be sensitive to any barriers migrant men may face within the maternity context including cultural, language or lack of understanding of maternity care [84]. More research is needed to explore migrant fathers’ experiences and to identify their needs with pregnancy and childbirth in order that interventions can be developed to support them.

### Strengths and limitations of review

Although this review refers to the pregnancy and childbirth experiences of fathers, most of the expectant fathers included in this review were non-migrant fathers with only 16 in total being migrant fathers. Nonetheless, this review has exposed the scarcity of research on the involvement of migrant fathers in pregnancy and childbirth care episodes and the lack of consideration to the type of migrant and how this may influence the father’s experience of pregnancy and childbirth.

## Conclusion

This review has revealed three main themes that influence fathers’ experiences of pregnancy and childbirth. These are: influence of society and health professionals; adjustment to a new life of fatherhood; and involvement in maternity. This review has demonstrated that there is a wealth of literature about the experiences of non-migrant fathers and that there are suggested similarities across various studies. However, there is a lack of data around migrant fathers. Data suggests that there may be some similar themes but there also appear to be concerns and issues in this heterogenous group. Hence, the literature focuses on non-migrant fathers’ experiences at the exclusion of migrant fathers. However, migrant fathers are also a heterogenous group with some men choosing to move to a new country and others being forced to migrate- including asylum seekers and refugees. Due to their life experiences, forced migrant fathers are in a more vulnerable position as compared to economic and educational migrants. There is a dearth of literature around migrant fathers’ experiences, and it is important that future research considers the heterogeneity of migrant fathers and how this influences their experiences of pregnancy and childbirth in their new country. From this, migrant fathers’ needs can be assessed, and appropriate interventions developed.

## Electronic supplementary material

Below is the link to the electronic supplementary material.

**Additional file 1: Appendix 1.** List of excluded studies in systematic review excluded studies with reasons

## Data Availability

The datasets generated or analysed during the current study are not publicly available due to this review being part of a PhD research. They are available from corresponding author upon reasonable request.
