# Supplementary material for: A systematic review and narrative synthesis of fathers’ (including migrant fathers’) experiences of pregnancy and childbirth
Source: BMC Pregnancy Childbirth. 2023 Apr 11;23:238. doi: 10.1186/s12884-023-05568-8 (PMC10088224; doi:10.1186/s12884-023-05568-8)
Supplement: Supplementary file 1 — Additional file 1: Appendix 1. List of excluded studies in systematic review excluded studies with reasons [file 12884_2023_5568_MOESM1_ESM.docx]

### Appendix 1: List of Excluded studies in Systematic Review

**Excluded Studies with reasons**

| **STUDY, COUNTRY** | **TITLE** | **REASON FOR EXCLUSION** |
| --- | --- | --- |
| **(Aarnio, Olsson et al. 2009)**  **MALAWI** | Male involvement in antenatal HIV counselling and testing: Exploring men's perceptions in rural Malawi | Setting not in high income country |
| **(An and Bang 2014)**  **SOUTH KOREA** | Effects of new born care education for first-time fathers on their knowledge and confidence in new born care at postpartum one month | Not published in English |
| **(Bergström, Kieler et al. 2009)**  **SWEDEN** | Effects of natural childbirth preparation versus standard antenatal education on epidural rates, experience of childbirth and parental stress in mothers and fathers: a randomised controlled multicentre trial | Not a normal childbirth experience |
| **(Bond 2010)**  **USA** | The missing link in MCH: Paternal involvement in pregnancy outcomes | Not a primary research |
| **(Buston 2010)**  **UK** | Experiences of, and attitudes towards, pregnancy and fatherhood amongst incarcerated young male offenders: findings from a qualitative study | Participants less than 18 years |
| **(Capogna, Camorcia et al. 2007)**  **ITALY** | Expectant fathers’ experience during labour with or without epidural analgesia | Study more than 10 years |
| **(Carter 2002)**  **GUATEMALA** | 'Because he loves me': Husbands' involvement in maternal health in Guatemala | Study more than 10 years |
| **(Chandler and Field 1997)**  **CANADA** | Becoming a father: First‐time fathers' experience of labour and delivery | Study more than 10 years |
| **(Chang, McNamara et al. 2006)**  **USA** | Identifying risk drinking in expectant fathers | Study more than 10 years |
| **(Clinton 1986)**  **USA** | Expectant fathers at risk for couvade | Study more than 10 years |
| **(Clinton 1987)**  **USA** | Physical and emotional responses of expectant fathers throughout pregnancy and the early postpartum period | Study more than 10 years |
| **(Cook 2000)**  **UK** | Mothers' and fathers' distress following childbirth: a discursive interactional perspective | Study more than 10 years |
| **(Côté-Arsenault 2016)**  **USA** | Have no regrets:” Parents’ experiences and developmental tasks in pregnancy with a lethal fetal diagnosis | Not a normal childbirth experience |
| **(Cronenwett 1974)**  **USA** | Fathers' responses to childbirth | Study more than 10 years |
| **(Dalton 2000)**  **UK** | Fathers' expectations and experiences of childbirth: implications for postnatal adjustment | Study more than 10 years |
| **(Danerek 2008)**  **SWEDEN** | A theoretical model of parents' experiences of threat of preterm birth in Sweden | Study more than 10 years |
| **(Dayton, Goletz et al. 2016)**  **USA** | Expectant fathers’ beliefs and expectations about fathering as they prepare to parent a new infant | About fatherhood and parenthood |
| **(Divney and Gordon 2016)**  **USA** | Stress and behavioral risk among young expectant couples | Not a normal childbirth experience |
| **(Etheridge 2014)**  **UK** | The experiences of fathers who found childbirth traumatic | Not a normal childbirth experience |
| **(Ferguson and Gates 2015)**  **UK** | Early intervention and holistic, relationship‐based practice with fathers: evidence from the work of the Family Nurse Partnership | About fatherhood and parenthood |
| **(Finnbogadóttir and Svalenius 2003)**  **SWEDEN** | Expectant first-time fathers’ experiences of pregnancy | Study more than 10 years |
| **(Frost 1997)**  **USA** | Postpartum distress in fathers: Predicting depressive symptoms, anxiety, and anger at one month postpartum | Study more than 10 years |
| **(Greenhalgh, Slade et al. 2000)**  **UK** | Fathers' coping style, antenatal preparation, and experiences of labor and the postpartum | Study more than 10 years |
| **(Gungor and Beji 2007)**  **TURKEY** | Effects of fathers’ attendance to labor and delivery on the experience of childbirth in Turkey | Study more than 10 years |
| **(Hallgreen, Kihlgren et al. 1999)**  **SWEDEN** | Swedish fathers' involvement in and experiences of childbirth preparation and childbirth | Study more than 10 years |
| **(Harvey 2010)**  **UK** | The experiences and perceptions of fathers attending the birth and immediate care of their baby | Not a normal childbirth experience |
| **(Hauari and Hollingworth 2009)**  **UK** | Understanding fathering: Masculinity, diversity and change | About fatherhood and parenthood |
| **(Department of Health 2011)**  **USA** | Parent's views on the maternity journey and early parenthood | Not a primary research |
| **(Johnson 2002)**  **UK** | The implications of unfulfilled expectations and perceived pressure to attend the birth on men's stress levels following birth attendance: a longitudinal study | Study more than 10 years |
| **(Johnson 2002)**  **UK** | An exploration of men's experience and role at childbirth | Study more than 10 years |
| **(Kowlessar, Fox et al. 2015)**  **UK** | First-time fathers’ experiences of parenting during the first year | About fatherhood and parenthood |
| **(Liebenau 2000)**  **UK** | Fathers' experiences of childbirth: an investigation into post-traumatic stress symptoms in first-time fathers and related factors | Study more than 10 years |
| **(Liebenberg 1969)**  **SWE** | Expectant fathers | Study more than 10 years |
| **(Lindberg and Öhrling 2007)**  **SWEDEN** | The birth of premature infants: experiences from the fathers’ perspective | Study more than 10 years |
| **(Lisy, Peters et al. 2016)**  **AUSTRALIA** | Provision of meaningful care at diagnosis, birth, and after stillbirth: A qualitative synthesis of parents' experiences | Not a normal childbirth experience |
| **(Lukesch 1977)**  **AUSTRIA** | The pregnancy experience according to expectant fathers | Study more than 10 years |
| **(Navarro, López et al. 2009)**  **MEXICO** | Paternity and health services. Qualitative research on men's experiences during pregnancy, delivery and postpartum of theirs partners | Setting not in high income country |
| **(Nichols 1993)**  **USA** | Paternal perspectives of the childbirth experience | Study more than 10 years |
| **(Ny, Plantin et al. 2007)**  **SWEDEN** | Middle Eastern mothers in Sweden, their experiences of the maternal health service and their partner's involvement | Study more than 10 years |
| **(Olin and Faxelid 2003)**  **SWEDEN** | Parents' needs to talk about their experiences of childbirth | Study more than 10 years |
| **(Plantin, Olykoya et al. 2011)**  **SWEDEN** | Positive health outcomes of fathers’ involvement in pregnancy and childbirth paternal support: a scope study literature review | Not a primary research |
| **(Premberg, Hellström et al. 2008)**  **SWEDEN** | Experiences of the first year as father | Study more than 10 years |
| **(Premberg and Lundgren 2006)**  **SWEDEN** | Fathers' experiences of childbirth education | Study more than 10 years |
| **(Redshaw and Henderson 2013)**  **UK** | Fathers’ engagement in pregnancy and childbirth: evidence from a national survey | Not a primary research |
| **(Reid, Wieck et al. 2017)**  **UK** | The Experiences of Fathers When Their Partners are Admitted with Their Infants to a Psychiatric Mother and Baby Unit | Not a normal childbirth experience |
| **(Roll and Cheater 2016)**  **UK** | Expectant parents’ views of factors influencing infant feeding decisions in the antenatal period: A systematic review | Not a primary research |
| **(Ross, Church et al. 2012)**  **TURKEY** | The perspectives of young men and their teenage partners on maternity and health services during pregnancy and early parenthood | Participants less than 18 years |
| **(Sahip and Turan 2007)**  **TURKEY** | Education for expectant fathers in workplaces in Turkey | Study more than 10 years |
| **(Salway, Chowbey et al. 2009)**  **UK** | Parenting in modern Britain: understanding the experiences of Asian fathers | About fatherhood and parenthood |
| **(Schoppe‐Sullivan, Kamp Dush et al. 2014)**  **USA** | Expectant fathers’ intuitive parenting: Associations with parent characteristics and postpartum positive engagement | About fatherhood and parenthood |
| **(Story, Sodzi-Tettey et al. 2016)**  **GHANA** | Male Involvement and Accommodation During Obstetric Emergencies in Rural Ghana: A Qualitative Analysis | Setting not in high income country |
| **(Tehrani, Bazzazian et al. 2015)**  **IRAN** | Pregnancy experiences of first-time fathers in Iran: A qualitative interview study | Setting not in high income country |
| **(Tikotzky, Sadeh et al. 2015)**  **ISRAEL** | Infant sleep development from 3 to 6 months postpartum: links with maternal sleep and paternal involvement | More than six weeks postpartum |
| **(Tyrer, Chase et al. 2005)**  **UK** | ‘Dealing with it’: Experiences of young fathers in and leaving care | Study more than 10 years |
| **(Vehviläinen-Julkunen and Liukkonen 1998)**  **FINLAND** | Fathers' experiences of childbirth | Study more than 10 years |
| **(Wee, Skouteris et al. 2015)**  **AUSTRALIA** | The inter-relationship between depressive, anxiety and stress symptoms in fathers during the antenatal period | Not a normal childbirth experience |
| **(Yargawa and Leonardi-Bee 2015)**  **UK** | Male involvement and maternal health outcomes: Systematic review and meta-analysis | Not a primary research |

Aarnio, P., et al. (2009). "Male involvement in antenatal HIV counseling and testing: Exploring men's perceptions in rural Malawi." AIDS Care **21**(12): 1537-1546.

An, H.-S. and K.-S. Bang (2014). "[Effects of newborn care education for first-time fathers on their knowledge and confidence in newborn care at postpartum one month]." Journal Of Korean Academy Of Nursing **44**(4): 428-436.

Bergström, M., et al. (2009). "Effects of natural childbirth preparation versus standard antenatal education on epidural rates, experience of childbirth and parental stress in mothers and fathers: a randomised controlled multicentre trial." International Journal of Obstetrics and Gynaecology **116**(9): 1167-1176.

Bond, M. J. (2010). "The missing link in MCH: Paternal involvement in pregnancy outcomes." American Journal of Men's Health **4**(4): 285-286.

Buston, K. M. (2010). "Experiences of, and attitudes towards, pregnancy and fatherhood amongst incarcerated young male offenders: findings from a qualitative study." Social Science & Medicine **71**(12): 2212-2218.

Capogna, G., et al. (2007). "Expectant fathers’ experience during labor with or without epidural analgesia." International Journal of Obstetrics Anesthesia **16**(2): 110-115.

Carter, M. W. (2002). "'Because he loves me': Husbands' involvement in maternal health in Guatemala." Culture, Health & Sexuality **4**(3): 259-279.

Chandler, S. and P. A. Field (1997). "Becoming a father: First‐time fathers' experience of labor and delivery." Nursing and Midwifery **42**(1): 17-24.

Chang, G., et al. (2006). "Identifying risk drinking in expectant fathers." Birth: Issues in Perinatal Care **33**(2): 110-116.

Clinton, J. F. (1986). "Expectant fathers at risk for couvade." Nursing research **35**(5): 290-295.

Clinton, J. F. (1987). "Physical and emotional responses of expectant fathers throughout pregnancy and the early postpartum period." International Journal of Nursing Studies **24**(1): 59-68.

Cook, M. (2000). Mothers' and fathers' distress following childbirth: a discursive interactional perspective, University of East London.

Côté-Arsenault, D., Denney-Koelsch, E. (2016). "“Have no regrets:” Parents’ experiences and developmental tasks in pregnancy with a lethal fetal diagnosis." Social Science and Medicine **154**: 100-109.

Cronenwett, L. R., Newmark, Lucy L. (1974). "Fathers' responses to childbirth." Nursing research.

Dalton, L. J. (2000). Fathers' expectations and experiences of childbirth: implications for postnatal adjustment, Open University.

Danerek, M., Dykes, Anna-Karin. (2008). "A theoretical model of parents' experiences of threat of preterm birth in Sweden." Midwifery **24**(4): 416-424.

Dayton, C. J., Buczkowski, Raelynn., Muzik, Maria.,, et al. (2016). "Expectant fathers’ beliefs and expectations about fathering as they prepare to parent a new infant." Social Work Research: 1-11.

Department of Health (2011). Parent's views on the maternity journey and early parenthood, The Stationery Office London.

Divney, A. and D. Gordon, Magriples, Urania., Kershaw, Trace. (2016). "Stress and behavioral risk among young expectant couples." Journal of Adolescence **53**: 34-44.

Etheridge, J. (2014). The experiences of fathers who found childbirth traumatic, University of Liverpool.

Ferguson, H. and P. Gates (2015). "Early intervention and holistic, relationship‐based practice with fathers: evidence from the work of the F amily N urse P artnership." Child, Family and Social Work **20**(1): 96-105.

Finnbogadóttir, H. and E. C. Svalenius, Persson, Eva K. (2003). "Expectant first-time fathers’ experiences of pregnancy." Midwifery **19**(2): 96-105.

Frost, L. A. (1997). Postpartum distress in fathers: Predicting depressive symptoms, anxiety, and anger at one month postpartum, ProQuest Information & Learning. **57:** 4706-4706.

Greenhalgh, R., et al. (2000). "Fathers' coping style, antenatal preparation, and experiences of labor and the postpartum." Birth (Berkeley, Calif.) **27**(3): 177-184.

Gungor, I. and N. K. Beji (2007). "Effects of fathers’ attendance to labor and delivery on the experience of childbirth in Turkey." Western Journal of Nursing Research **29**(2): 213-231.

Hallgreen, A., et al. (1999). "Swedish fathers' involvement in and experiences of childbirth preparation and childbirth." Midwifery **15**(1): 6-15.

Harvey, M. E. (2010). The experiences and perceptions of fathers attending the birth and immediate care of their baby, Aston University.

Hauari, H. and K. Hollingworth (2009). "Understanding fathering: Masculinity, diversity and change."

Johnson, M. P. (2002). "An exploration of men's experience and role at childbirth." Men's Studies **10**(2): 165-182.

Johnson, M. P. (2002). "The implications of unfulfilled expectations and perceived pressure to attend the birth on men's stress levels following birth attendance: a longitudinal study." Journal Of Psychosomatic Obstetrics And Gynaecology **23**(3): 173-182.

Kowlessar, O., et al. (2015). "First-time fathers’ experiences of parenting during the first year." Journal of Reproductive and Infant Psychology **33**(1): 4-14.

Liebenau, A. (2000). Fathers' experiences of childbirth: an investigation into post-traumatic stress symptoms in first-time fathers and related factors, University of Exeter.

Liebenberg, B. (1969). "Expectant fathers." Child & Family **8**(3): 265-277.

Lindberg, B., Axelsson, Karin., and K. Öhrling (2007). "The birth of premature infants: experiences from the fathers’ perspective." Neonatal Nursing **13**(4): 142-149.

Lisy, K., et al. (2016). "Provision of meaningful care at diagnosis, birth, and after stillbirth: A qualitative synthesis of parents' experiences." Birth: Issues in Perinatal Care **43**(1): 6-19.

Lukesch, H. (1977). "The pregnancy experience according to expectant fathers." Psychologie und Praxis **21**(3): 123-131.

Navarro, G. M., et al. (2009). "Paternity and health services. Qualitative research on men's experiences during pregnancy, delivery and postpartum of theirs partners." Revista Espanola De Salud Publica **83**(2): 267-278.

Nichols, M. R. (1993). "Paternal perspectives of the childbirth experience." Maternal Child Nursing.

Ny, P., et al. (2007). "Middle Eastern mothers in Sweden, their experiences of the maternal health service and their partner's involvement." Reproductive Health **4**.

Olin, R. M. and E. Faxelid (2003). "Parents' needs to talk about their experiences of childbirth." Scandinavian Journal of Caring Sciences **17**(2): 153-159.

Plantin, L., et al. (2011). "Positive health outcomes of fathers’ involvment in pregnancy and childbirth paternal support: a scope study literature review."

Premberg, Å., et al. (2008). "Experiences of the first year as father." Scand J Caring Sci **22**(1): 56-63.

Premberg, A. and I. Lundgren (2006). "Fathers' experiences of childbirth education." Journal of Perinatal Education **15**(2): 21.

Redshaw, M. and J. Henderson (2013). "Fathers’ engagement in pregnancy and childbirth: evidence from a national survey." BMC pregnancy and childbirth **13**(1): 70.

Reid, H., et al. (2017). "The Experiences of Fathers When Their Partners are Admitted with Their Infants to a Psychiatric Mother and Baby Unit." Clinical Psychology & Psychotherapy **24**(4): 919-931.

Roll, C. L. and F. Cheater (2016). "Expectant parents’ views of factors influencing infant feeding decisions in the antenatal period: A systematic review." International Journal of Nursing Studies **60**: 145-155.

Ross, N. J., et al. (2012). "The perspectives of young men and their teenage partners on maternity and health services during pregnancy and early parenthood." Children and Society **26**(4): 304-315.

Sahip, Y. and J. M. Turan (2007). "Education for expectant fathers in workplaces in Turkey." Journal Of Biosocial Science **39**(6): 843-860.

Salway, S., et al. (2009). Parenting in moden Britain: understanding the experiences of Asian fathers, Joseph Rowntree Foundation.

Schoppe‐Sullivan, S. J., Altenburger, Lauren E., Settle, Theresa A., , et al. (2014). "Expectant fathers’ intuitive parenting: Associations with parent characteristics and postpartum positive engagement." Infant Mental Health Journal **35**(5): 409-421.

Story, W. T., Barrington, Clare., Fordham, Corinne.,, et al. (2016). "Male Involvement and Accommodation During Obstetric Emergencies in Rural Ghana: A Qualitative Analysis." International Perspectives on Sexual and Reproductive Health **42**(4): 211-219.

Tehrani, S. G., et al. (2015). "Pregnancy experiences of first-time fathers in Iran: A qualitative interview study." Iranian Red Crescent Medical Journal **17**(2).

Tikotzky, L., et al. (2015). "Infant sleep development from 3 to 6 months postpartum: links with maternal sleep and paternal involvement." Monographs Of The Society For Research In Child Development **80**(1): 107-124.

Tyrer, P., et al. (2005). "‘Dealing with it’: Experiences of young fathers in and leaving care." British Journal of Social Work **35**(7): 1107-1121.

Vehviläinen-Julkunen, K. and A. Liukkonen (1998). "Fathers' experiences of childbirth." J Midwifery **14**(1): 10-17.

Wee, K. Y., et al. (2015). "The inter-relationship between depressive, anxiety and stress symptoms in fathers during the antenatal period." Journal of Reproductive and Infant Psychology **33**(4): 359-373.

Yargawa, J. and J. Leonardi-Bee (2015). "Male involvement and maternal health outcomes: Systematic review and meta-analysis." Journal of Epidemiology and Community Health **69**(6): 604-612.
